# Application of Silsesquioxanes in the Preparation of Polyolefin-Based Materials

**DOI:** 10.3390/ma16051876

**Published:** 2023-02-24

**Authors:** Marzena Białek, Krystyna Czaja

**Affiliations:** Institute of Chemistry, University of Opole, Oleska 48, 45-052 Opole, Poland

**Keywords:** polyolefins, olefin, POSS, polymerization, copolymerization, POSS-comonomer, catalyst, transition metal–silsesquioxane complex, composite, siloxane–silsesquioxane resin

## Abstract

This paper is a review of studies on the use of the polyhedral oligomeric silsesquioxanes (POSS) of various structures in the synthesis of polyolefins and the modification of their properties, namely: (1) components of organometallic catalytic systems for the polymerization of olefins, (2) comonomers in the copolymerization with ethylene, and (3) fillers in composites based on polyolefins. In addition, studies on the use of new silicon compounds, i.e., siloxane–silsesquioxane resins, as fillers for composites based on polyolefins are presented. The authors dedicate this paper to Professor Bogdan Marciniec on the occasion of his jubilee.

## 1. Introduction

After a stagnation in 2020 due to the COVID-19 pandemic, global plastics production increased to 390.7 million tons in 2021. The largest part of the world’s production is made up of polyolefins. It is estimated that approximately 105 million tons of polyethylene (PE) and 75 million tons of polypropylene (PP) were produced worldwide in 2021 in total, covering 46.2% of global plastics production [1]. Meanwhile, in the 1950s, the market share of polyolefins was negligible. A rapid increase in their production was initiated by the discovery of organometallic catalytic systems for low-pressure polymerization according to coordination mechanism. Since then, development of organometallic catalysts in terms of their activity, better control of polymers microstructure, and possibility to copolymerize olefin with a wider range of vinyl monomers can be observed.

Many beneficial properties of polyolefins, such as chemical inertness, mechanical properties, lack of potential toxicity, good processability, and possibility of reuse by recycling, mean that only a few materials can match their versatility and economy. However, despite many advantages, the limited thermal resistance and high susceptibility to flammability of polyolefins worsens the safety of their use and makes it necessary to develop new polyolefin materials that meet the requirements for more demanding applications [2]. In this respect, copolymerization of olefins with appropriately functionalized silsesquioxanes, and, in particular, the use of POSS as fillers in polyolefin composites creates significant opportunities. The latest trends in this field are related to the development of hybrid (i.e., organic–inorganic) materials [3,4,5,6,7,8,9,10].

Special attention has been focused on polyhedral oligomeric silsesquioxanes (POSS) with the general formula (RSiO_3/2_)_n_ built of a silicon–oxygen core in the form of a cage and reactive and/or nonreactive substituents [3]. One of the most important features of POSS compounds is the outstanding possibility of modifying their structure by changing the silicon–oxygen core, and, above all, the type of substituents attached to each corner silicon atom, which may be hydrocarbon in nature or may contain polar and functional groups. This allows for significant differentiation of their properties, which contributes to intensive development of research on the synthesis and application of silsesquioxane derivatives [3,4,11].

Herein, we present the recent achievements in the use of silsesquioxanes in the synthesis and modification of polyolefins. The review is divided into three main sections, which correspond to the three main research directions in the field of polyolefins. Research on the use of POSS-containing catalysts in olefin polymerization are discussed in Chapter 2, below. The development in the research on the copolymerization of alkenes with mono- or multi-alkenyl-POSS compounds, carried out by coordination polymerization, is described in Chapter 3. In particular, the effect of the catalyst type and the structure of the POSS comonomer on the amount and the manner of comonomer incorporation into polyethylene macromolecules in connection with the properties of the obtained copolymers, including their thermal properties and oxidation resistance, is discussed. The state of art on POSS-containing polyolefin composites and also composites filled with siloxane–silsesquioxane resins are characterized in Chapter 4 of this article. First of all, the influence of the incorporation of POSS nanoparticles into a polyolefin matrix via physical blending on the structure and the mechanical, thermal, rheological, and morphological properties of obtained nanocomposites are shown. The review includes our research in these areas, which we carried out in cooperation with the research group of prof. Bogdan Marciniec, which is specialized in the synthesis of silsesquioxane compounds with various structures.

## 2. Silsesquioxane Moiety in Precatalysts for Olefin Polymerization

Metal-containing silsesquioxanes and other silsesquioxanes derivatives are extensively used in the field of catalysis. Compounds of that type have been successfully employed as catalysts in various chemical reactions, such as metathesis [12,13], epoxidation [14,15,16,17,18], esterification [19,20], reduction [21,22,23], and even hydrogen production through autothermal reforming of methane [24]. A number of works have also been devoted to the use of POSS in the catalytic synthesis of polyolefins. In this process, POSS are used as components of both heterogeneous and homogeneous catalytic systems. In the former, they usually act as a carrier modifier, and, in the latter, they make a ligand in transition metal complex or, due to the presence of alkyl or aryl groups linked to silsesquioxane cages which make the molecule soluble in organic solvents, are used for the preparation of homogeneous analogs of supported catalysts.

### 2.1. POSS as a Carrier Modifier and Homogeneous Support

Silsesquioxanes were applied to modify both silica support for metallocene and post-metallocene catalysts and magnesium chloride used as a support in Ziegler–Natta catalysts. Galland and others immobilized metallocene, (nBuCp)_2_ZrCl_2_, on a polyhedral oligomeric silsesquioxane-modified silica [25,26]. The studies, which included the effect of the POSS/SiO_2_ and Zr/SiO_2_ ratios on catalyst performance, revealed that POSS, acting as a horizontal spacer of metallocene molecules adsorbed on the silica surface, increased the catalyst activity. At the optimum range of POSS and metallocene concentrations on the carrier catalyst, activities were comparable to those of a homogeneous system [26]. Li et al. employed POSS to functionalize silica pretreated with methylaluminoxane for immobilization of the fluorinated bis(phenoxyimine)titanium complex (FI catalyst) [27,28]. In this case, POSS also served as horizontal spacers between active sites leading to more active catalysts than that supported on the POSS-free carrier. Moreover, the ultra-high molecular weight polyethylene (UHMWPE) produced by FI/POSS/SiO_2_ systems showed a weakly entangled (disentangled) state. Lower entangled density advantageously affects some properties of polyolefins (e.g., crystallinity and mechanical properties) and is favorable from the point of view of their processing [29,30]. For this reason, research has also been undertaken on POSS-modified Ziegler–Natta catalysts for synthesis of ultra-high molecular weight PE with reduced entanglement density [30,31,32,33]. It has been shown that the properties of the catalytic system are significantly influenced by the content of hydroxyl groups in the POSS applied. All POSS-modified catalysts exhibited higher activities and produced UHMWPE with reduced entangled states compared to the POSS-free catalyst; nevertheless, the optimal effect of reducing entanglement was obtained with the catalyst supported on MgCl_2_ modified with POSS without hydroxyl groups [31].

**Figure 1:**
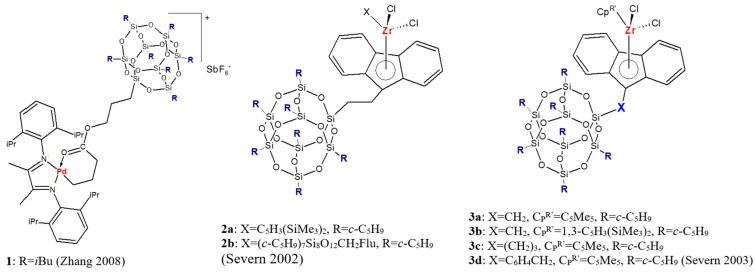
Homogeneous analogs of supported catalysts with silsesquioxane moiety used in olefin polymerization [34,35,36].

There are also some examples of using POSS molecules for preparation of homogeneous analogs of supported catalysts (Figure 1). Zhang and Ye synthesized a homogeneous POSS-supported palladium catalyst **1** using a Pd–diimine complex and a completely condensed POSS macromonomer bearing an acrylate functionality [34]. Such a catalyst was able to initiate “living” ethylene polymerization and produce oil-like liquid polymers with chains end-capped with a POSS nanoparticle. To form supported zirconocene catalysts, sequential synthesis of a silsesquioxane-tethered fluorenyl ligand followed by ligand deprotonation and reaction with zirconium precursors was used [35,36]. Prepared zirconocenes **2a**, **2b**, and **3a–3d** activated with methylalumoxane (MAO) were found to generate active ethylene polymerization catalysts; however, their activity was lower than the activity of corresponding non-silsesquioxane catalysts.

### 2.2. POSS as a Ligand in Transition Metal Complexes

Incompletely condensed silsesquioxanes with different numbers of silanol groups and completely condensed silsesquioxanes with OH side group were successfully applied as single or additional ancillary ligands for the main groups and transition metals [37]. Complexes of that type, as mentioned before, were employed as catalysts for various chemical reactions, including olefin polymerization precatalysts based on transition metals of groups four to six of the periodic table [38]. However, it should be noted that studies concerning catalytic olefin polymerization have limited scope and concern mainly polyethylene synthesis and, to a small extent, the polymerization of higher 1-olefins. Moreover, in many cases, such complexes were investigated in olefin polymerization as models of catalysts immobilized on silica.

**Figure 2:**
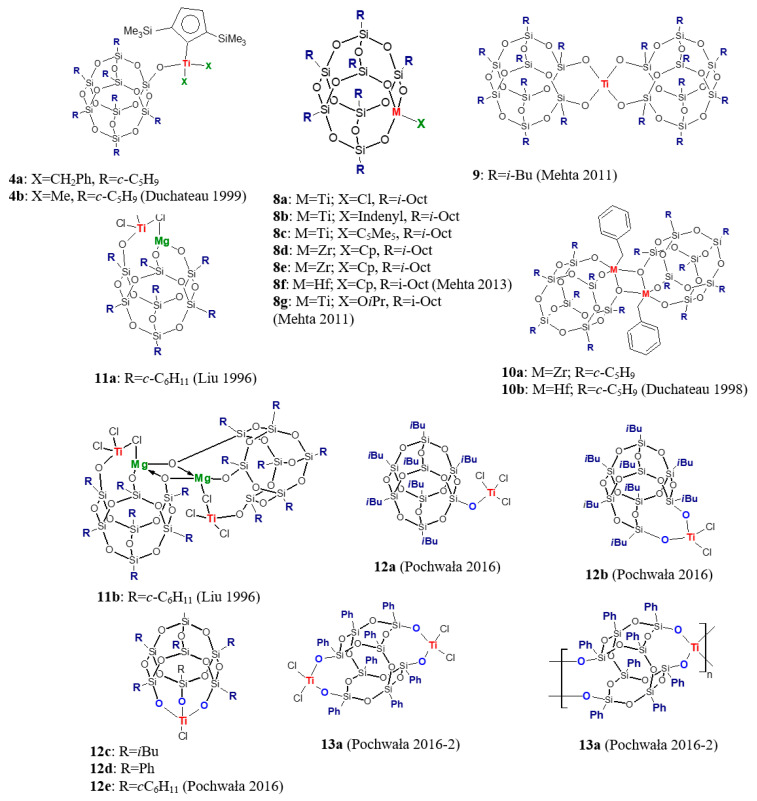
The fourth group transition metals complexes bearing silsesquioxane ligands used in olefin polymerization [39,43,44,45,46,47,48].

Silsesquioxane complexes of the fourth group transition metals, especially titanium, are most commonly used in olefin polymerization. Their structures are very diverse. They differ in the denticity of the POSS ligand, the type of non-reactive substituents, and the presence of atoms of elements other than silicon and oxygen in the POSS structure. Moreover, monomeric and dimeric complexes can be distinguished among them and complexes bearing, in addition to silsesquioxane, another ancillary ligand: cyclopentadienyl, substituted cyclopentadienyl, or indenyl (Figure 2). Duchateau and others [39] synthesized half-sandwich titanium silsesquioxane bis(alkyl) complexes **4a** and **4b**, which, in conjunction with B(C_6_F_5_)_3_, form active ethylene polymerization catalysts showing activity equal to 880 and 840 kg/(mol·h·atm), respectively. Moreover, catalyst **4a**/B(C_6_F_5_)_3_ was found to polymerize 1-hexene into atactic poly(1-hexene), although with low activity [39]. Other titanium half-sandwich complexes Cp’’[(c-C_5_H_9_)_7_Si_7_O_10_(OSiMe_2_O)]Ti(CH_2_Ph)_2_ (**5a**) and Cp’’[(c-C_6_H_11_)_7_Si_7_O_10_(O{SiMe_2_O}_2_)]Ti(CH_2_Ph)_2_ (**5b**) [40] were also tested in 1-hexene polymerization. Their activity was an order of magnitude lower, after activation with B(C_6_F_5_)_3_, than when [Ph_3_C][B(C_6_F_5_)_4_] were used as cocatalysts. Moreover, it was shown that cationic titanium species formed by reacting **5a** and **5b** with B(C_6_F_5_)_3_ are stable for a long time, in contrast to active species formed in **4a**/B(C_6_F_5_)_3_ based on the silsesquioxane with isolated silanol [40]. Titanium complexes Cp”[(*c*-C_5_H_9_)_7_Si_8_O_13_]TiCl_2_ (**6a**) and Cp”[(*c*-C_5_H_9_)_7_Si_8_O_13_]_2_TiCl (**6b**) (Cp” = η^5^-1,3-C_5_H_3_(SiMe_3_)_2_) [41] in combination with methylalumoxane also produced active catalysts for ethylene polymerization, with activity of 5.7 kg/(mmol·h). However, facile activation of **6b** with MAO raised questions about the stability of metallasilsesquioxanes in reaction with aluminum alkyls. Zirconocene complexes with silsesquioxane moiety [{(*c*-C_5_H_9_)_7_Si_8_O_12_O}_2_Zr(η^5^-C_5_H_5_)_2_] (**7a**) and [(*c*-C_5_H_9_)_7_Si_7_O_9_(OSiMe_3_)O_2_}- Zr(η^5^-C_5_H_5_)_2_] (**7b**) when combined with MAO were approximately as active as ZrCl_2_(η^5^-C_5_H_5_)_2_] [42].

Mehta et al., using the triol derivative (*i*-Octyl)_7_Si_7_O_9_(OH)_3_, synthesized a series of group 4 metallasilsesquioxanes **8a**–**8g** with additional X ligand (X = Cl, Cp, C_5_Me_5,_ Indenyl, O*i*Pr) and evaluated them in ethylene polymerization [43,44]. The research focused on the effect of alkylaluminium cocatalyst (Et_3_Al_2_Cl_3_, Et_2_AlCl, Et_3_Al, MAO) and reaction conditions (Al/M molar ratio, reaction temperature, ethylene pressure) on catalyst activity and polyethylene properties. Investigated complexes turned out to be most active in combination with ethylaluminum sesquichloride at high temperature (100 °C) and produced linear low molecular weight polyethylene with narrow dispersity. Its properties were comparable to those of commercial micronized PE waxes. A similar product was obtained in the presence of a dimeric titanium complex **9 [44]**. Dimeric zirconium (**10a**) and hafnium (**10b**) complexes, synthesized in reaction of M(CH_2_Ph)_4_ (M = Zr, Hf) with (*c*-C_5_H_9_)_7_Si_7_O_9_(OH)_3_, after activation with B(C_6_F_5_)_3_ afforded the cationic mono(benzyl) complexes, which produce PE with activity equal to 2400 and 4800 g/(mmol·h), respectively. Moreover, complexes **10a** and **10b** were found to polymerize ethylene even without cocatalyst, though with low activity [45]. Liu reported synthesis and characterization of a bimetallic [(*c*-C_6_H_11_)_7_(Si_7_O_12_)MgTiCl_3_]*_n_* (*n* = 1, 2) precatalyst, which exists as a mixture of monomeric **11a** and dimeric **11b** form in the mole ratio of 3:1 [46]. With triethylaluminium as cocatalyst at 90 °C and in the presence of hydrogen it polymerized ethylene with higher activity than a typical commercial Ti/Mg/SiO_2_-supported catalyst having the same magnesium and titanium content (110.8 and 60.0 kg PE/(g_Ti_·h), respectively). Produced polyethylene was characterized by high molecular weight, *M_w_* = 140,000 g/mol, and *M_w_/M_n_* = 5.5.

Due to the wide variety of structures of four group transition metal–silsesquioxane compounds employed in olefin polymerization and the diverse polymerization conditions used by different research groups, it was difficult to draw any general conclusions about the relationship between catalytic system structure, its activity, and the properties of the polymerization products. Thus, we decided to conduct a systematic study on the effects of the structure of titanium silsesquioxane complexes, types of cocatalyst, and reaction conditions on the catalysts activity in ethylene and 1-octene polymerization [47,48]. The studied complexes (**12a**–**12e**) differed in the type of nonreactive substituents bonded to the inorganic oxygen–silicon cage (*i*Bu, Ph, *c*C_6_H_11_), ligand denticity (metal bound via one, two, and three oxygen atoms) and amount of chlorine in their structure. It was shown that all investigated complexes exhibited the highest activity in ethylene polymerization in conjunction with Et_2_AlCl as a cocatalyst (Et_2_AlCl > EtAlCl_2_ > Et_3_Al >> MAO). In turn, their activity (after activation with the most effective cocatalyst) varies, as follows, depending on the structure of the complex: **12b** > **12a** ≥ **12e** ≥ **12c** > **12d**. It means that the complex with bidentate POSS ligand displayed the highest activity. It produced 110.30 kg/(mol_Ti_·0.5 h) at 80 °C. Our studies also demonstrated that properties of obtained polymers are strongly influenced by the type of cocatalyst used. Complexes activated with Et_2_AlCl and EtAlCl_2_ produced low molecular weight polyethylenes with bimodal (Et_2_AlCl) or trimodal (EtAlCl_2_) molecular weight distribution, whereas using Et_3_Al led to polyethylene with high molecular weight (*M_w_* = 447 × 10^3^ g/mol) and low dispersity (*M_w_/M_n_* = 2.7). Moreover, ligand denticity is the other factor having a strong impact on the molecular weight of the produced polyethylene. *M_w_* of PE decreased as ligand denticity increased: 106,000 g/mol (**12a**/Et_2_AlCl) > 38,000 g/mol (**12b**/Et_2_AlCl) > 490 g/mol (**12c**/Et_2_AlCl) [47]. Bimetallic complex **13a** and its polymeric counterpart **13b** activated by diethylaluminium chloride and ethylaluminium dichloride were found to polymerize ethylene as well [48]. Complex **13a** showed higher activity (about 300%) than **13b**, and the influence of the type of cocatalyst on their activity and product properties turned out to be the same as in the case of complexes **12a**–**12e**. Selected titanium catalysts (**12a**/Et_2_AlCl and **12b**/EtAlCl_2_) were used in ethylene/1-octene copolymerization [47]. It was confirmed that they produce copolymers with a low level of comonomer incorporation, which was equal to 0.4–1.0%mol.

A literature review indicated a lack of information on the use of silsesquioxane complexes in higher 1-olefin polymerization (except for **4a**, **5a** and **5b** which, however, contained an additional cyclopentadienyl ligand). This motivated us to conduct extensive research on 1-octene polymerization [47,48]. Monometallic complexes **12a**–**12e** and bimetallic complex **13a** were used in polymerization after activation with various cocatalyst, starting from Et_2_AlCl through MAO and boron compounds ([Ph_3_C][B(C_6_F_5_)_4_], B(C_6_F_5_)_3_, [PhNMeH][B(C_6_F_5_)_4_]) in combination with (*i*Bu)_3_Al. Neither complex was active in this reaction when activated with a simple organoaluminum compound, and monomer conversion in the presence of other catalytic systems did not exceed 20.1%. Complexes **12c**–**12e,** having tridentate ligands, and bimetallic complex **13a** showed the highest activity in combination with MAO, while complexes **12a** and **12b** were not active in conjunction with this cocatalyst. Efficiency of the boron containing cocatalysts, regardless of the structure of the complex used, increased in series B(C_6_F_5_)_3_ < [PhNMeH][B(C_6_F_5_)_4_] < [Ph_3_C][B(C_6_F_5_)_4_]. The influence of the type of cocatalyst on the molecular weight of the polymers was opposite. The highest and the lowest molecular weight have polymers produced by complexes activated by B(C_6_F_5_)_4_ and [Ph_3_C][B(C_6_F_5_)_4_], respectively. For example 27,300 g/mol, 8200 g/mol, and 5800 g/mol had polymers produced in sequence by **12b**/B(C_6_F_5_)_4_, **12b**/[PhNMeH][B(C_6_F_5_)_4_] and **12b**/[Ph_3_C][B(C_6_F_5_)_4_]. In addition, it was found that the increase in the number of donor atoms in the ligand structure increased the activity of the catalyst (**12a** < **12b** < **12c**) and also resulted in a decrease in molecular weight of poly(1-octene). Microstructure analysis revealed that produced poly(1-olefins) were moderately isotactic, with *mmmm* pentad ranging from 44 to 74%, and possessed various end-groups suggesting involvement of different chain terminations after the previous 1,2- or 2,1-insertion of a monomer molecule [47,48].

**Figure 3:**
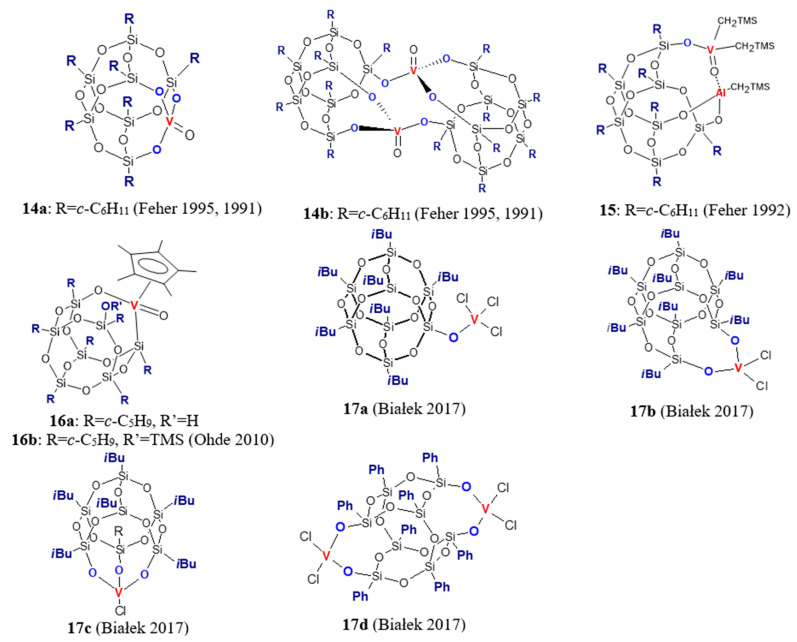
Silsesquioxane complexes of vanadium used in olefin polymerization [49,50,51,52,53].

**Figure 4:**
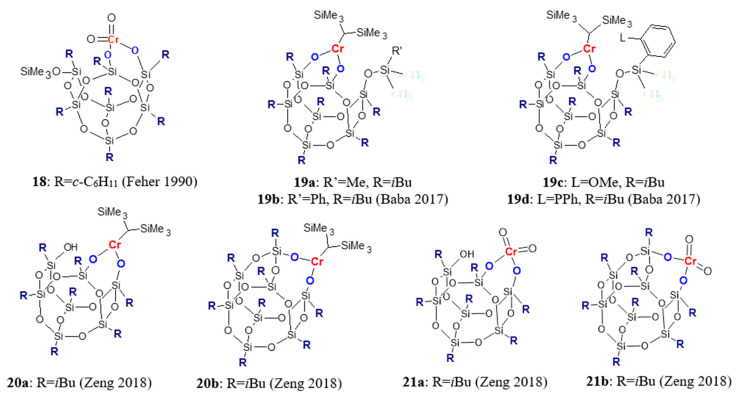
Silsesquioxane complexes of chromium used in olefin polymerization [54,55,56].

Silsesquioxane complexes of the fifth (Figure 3) and sixth (Figure 4) group transition metals for olefin polymerization are rather rare in literature, although the first information about vanadium and chromium complexes appeared as early as the early nineties of the last century. Feher et al. synthesized an oxovanadium–silsesquioxane complex (*c*-C_6_H_11_)_7_Si_7_O_9_(O_3_V = O), which, in solution, exists as a mixture of monomeric (**14a**) and dimeric (**14b**) forms [49,50]. Its activation with Me_3_Al (1–5 equiv/V) gave an active catalyst (the dimer **14b** is not directly involved but is a precursor to **14a** [49]) that produced linear polyethylene with *M_w_* equal to 47,900 g/mol, *M_w_/M_n_* = 2.28, and a high melting temperature of 131.6 °C [50]. Further research showed that oxo-vanadium(V) alkyl complex **15** also polymerized ethylene to polyethylene with similar properties, though its dispersity was larger (*M_w_/M_n_* = 5.72) [51]. Among vanadium silsesquioxane complexes, similarly as among the forth group transition metals complexes, there are those containing a cyclopentadienyl co-ligand. Complexes **16a** and **16b** activated by PMAO (polymethylaluminoxane free of trimethylaluminum) and ethylaluminiumdichloride were found to polymerize ethylene at atmospheric pressure with activity of 74–190 kg/(mol_V_·h) [52]. No clear trends for the effects of complex structure and the cocatalyst type on the activity were observed. On the other hand, the molecular weight and dispersity of the produced polymers were dependent on cocatalyst type. Ultra-high molecular weight polyethylenes with *M_w_* around 2·10^6^ g/mol and *M_w_/M_n_* around 5.8 were produced in the presence of PMAO, whereas EtAlCl_2_ led to products with lower *M*_w_ and dispersity (*M_w_* ≈ 6.5·10^5^ g/mol, *M_w_/M_n_* = 2.9).

Our studies involved complexes **17a**–**17d** [53]. To our knowledge, these are the first vanadium silsesquioxane complexes with VCl moiety used in olefin polymerization. The complexes **17a**–**17d** were employed in ethylene polymerization in conjunction with a different cocatalyst (Et_2_AlCl, EtAlCl_2_, MAO and (*i*Bu)_3_Al/[Ph_3_C][B(C_6_F_5_)_4_]. They turned out to be highly active after activation with EtAlCl_2_ (up to 8610.0 kg/(mol_V_·0.5 h)). In conjunction with other cocatalysts, their activity was lower and ranged from 58 kg/(mol_V_·0.5 h) to 345.7 kg/(mol_V_·0.5 h). Moreover, the efficiency of vanadium complexes activated by EtAlCl_2_ increased with increasing denticity of silsesquioxane ligand (**17a** < **17b** < **17c**), and bimetallic complex **17d** was most active. It was also shown that synthesized polyethylenes have high to ultra-high molecular weights (*M_w_* = 370 × 10^3^–4423 × 10^3^ g/mol) depending on the cocatalyst used and the reaction conditions. Unlike the titanium counterparts, all vanadium complexes, regardless of the type of cocatalyst used, produced polymers with unimodal molecular weight distribution. The results of the copolymerization test, which was carried out for **17d**/EtAlCl_2_, revealed considerable compositional heterogeneity of the produced copolymer and moderate incorporation of 1-octene comonomer (2.2 mol%).

Although vanadium–silsesquioxanes have never been used before in 1-olefin polymerization, we employed complexes **17a**–**17c** for the synthesis of poly(1-octene) [53]. The activity of all complexes depended on the type of cocatalyst used, as follows: MAO *>* [Ph_3_C][C(C_6_B_5_)_4_] *>* B(C_6_F_5_)_3_ *>* [PhNMe_2_H][B(C_6_F_5_)_4_] *>* Et_2_AlCl. Produced poly(1-octenes) were in the form of sticky solid material or viscous oil and were moderately isotactic (*mmmm* = 51–64%). Further studies showed that the polymer with the highest isotactic pentad content, 75%, can be obtained with **17d**/(*i*Bu)_3_Al/[Ph_3_C][B(C_6_F_5_)_4_] [53].

Chromium–silsesquioxane compounds (Figure 4) have been studied in the polymerization of ethylene as homogeneous models of Philips-type catalysts for ethylene polymerization [54,55,56]. Feher and Blanski synthesized **18** and found that it polymerized ethylene at 1 atm pressure after addition of 1.5–10 equivalent of Me_3_Al and produced linear polyethylene with *M_w_* = 30.7 × 10^3^ g/mol [54]. In much later work [56], chromium–silsesquioxane compounds **19a**–**19d** were obtained by reaction of Cr[CH(SiMe_3_)_2_]_3_ with functionalized POSS: R_7_Si_7_O_9_(OH)_2_(OSiMe_2_R’) where R = *i*Bu, R’ = Me, Ph, C_6_H_4_(OMe-2), C_6_H_4_(PPh_2–_2). It was shown that catalyst activity and the molecular weight of polyethylene were sensitive to the coordination environment of the chromium center. The activity of chromium complexes towards ethylene polymerization in the presence of (*i*Bu)_3_Al at 60 °C and at 0.5 MPa ranged from about 30 to about 60 kg/(mol_Cr_·h), and it was dependent on the modification of the POSS cage as follows: **19b** < **19c** < **19a** < **19d**. Complexes **19a** and **19b** at 50 °C gave polyethylene with a very high molecular weight (*M_w_* = 1.8·10^6^ and 3.3·10^6^ g/mol), with a rather wide molecular weight distribution (MWD) due to slight tailing on the low molecular side. At the same reaction condition, complexes **19c** and **19d** produced bimodal polyethylene with lower *M_w_*. The effect of the POSS cage structure and the chromium oxidation state on ethylene polymerization was presented in [55]. Complexes **20a**, **20b**, and **21a**, **21b** were synthesized in the reaction of POSS-triol and POSS-diol with Cr(III) (Cr[CH(SiMe_3_)_2_]_3_) and Cr(VI) (CrO_3_) compounds, respectively. Tri-*n*-octylaluminum and triisobutylaluminium modified with butylated hydroxytoluene (TIBA-BHT) were used as cocatalysts. It was revealed that all investigated complexes were less active than the appropriate chromium compounds supported on SiO_2_, and that the Cr oxidation state has an important effect on the behavior of the catalyst. Complexes containing Cr(VI) exhibited higher activities (up to 523 g/(g_cat_·h)) than trivalent ones (up to 64 g/(g_cat_·h)). Produced polyethylenes had bimodal molecular weight distributions.

## 3. Copolymerization of Ethylene with Alkenylsilsesquioxanes over Organometallic Catalysts

**Figure 5:**
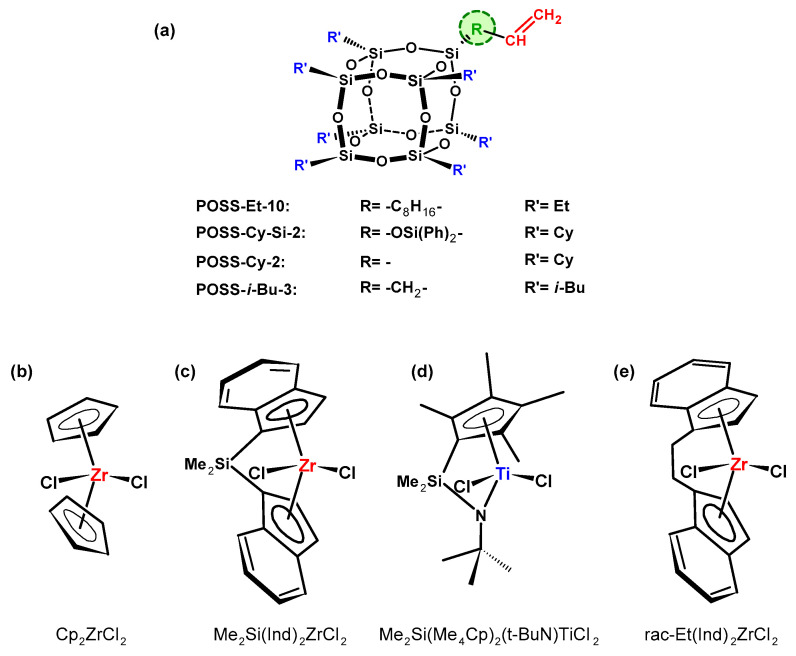
Structure of monoalkenylsilesquioxane comonomers (**a**) copolymerized with ethylene over metallocene catalysis systems (**b**–**e**) [68,69,70].

Hybrid (co)polymers containing polyhedral oligomeric silsesquioxanes have attracted great interest in recent years. Literature data show that functionalized silsesquioxanes, both monofunctional and multifunctional, such as styryl-POSS, methacrylate-POSS, norbornyl-POSS, amine-POSS, hydroxyl-POSS, epoxy-POSS, phenolic-POSS, benzoxazine-POSS, and alkenyl-POSS have been used as monomers in homo- or copolymerization [4,8,9,10,57]. They were (co)polymerized according to various mechanisms, including polycondensation [58,59,60], ring opening metathesis polymerization (ROMP) [61,62,63,64], or atom transfer radical polymerization (ATRP) [65,66,67]. However, there is only limited literature data [68,69,70] concerning the coordination (co)polymerization of selected monoalkenylsilsesquioxanes (Figure 5a) catalyzed with the use of only metallocene catalysts (Figure 5b–e).

The first literature report related to homopolymerization of POSS and copolymerization of POSS with ethylene or propylene was published by Tsuhida et al. [68]. The POSS-Et-10 comonomer (Figure 5a) was homopolymerized and copolymerized with ethylene or propylene. The polymerization was carried out over the metallocene complexes: Cp_2_ZrCl_2_, Me_2_Si(Ind)_2_ZrCl_2_, and Me_2_Si[(Me_4_Cp)(*t*-BuN)]TiCl_2_ (Figure 5b–d) activated with methylaluminoxane (MAO). It was found that during homopolymerization of POSS, only di- or trimers were obtained. The copolymer with highest POSS content of 24.5 wt.% was synthesized by Me_2_Si[(Me_4_Cp)(*t*-BuN)]TiCl_2_ complex (Figure 5d). Additionally, it turned out that the POSS comonomer caused a decrease in molecular weight of the obtained copolymers in comparison with neat polyolefins, and, therefore, took part in the termination reaction of macromolecules. Moreover, incorporation of POSS into the polymer chain resulted in slightly improved thermal stability (decomposition temperature, *T_d_*, of copolymers increased by 13 °C) in comparison with neat polyethylene and polypropylene.

Zhang et al. [69] reported ethylene copolymerization with different structures of monoalkenylsilsesquioxanes: POSS-Cy-2, POSS-Cy-Si-2, and POSS-*i*-Bu-3 (Figure 5a) over *rac*-Et(Ind)_2_ZrCl_2_, activated with modified methylaluminoxane (MMAO) (Figure 5e). The highest POSS content incorporated into the polymer chain was observed in case of ethylene copolymer with POSS-Cy-2 and POSS-Cy-Si-2. The value of *T_d_* for copolymers increased with increasing POSS content in the polymer chain by 5–23 °C in comparison with neat polyethylene. Additionally, POSS-Cy-2 and POSS-*i*-Bu-3 comonomers (Figure 5a) were also copolymerized with propylene [70] with the use of the *rac*-Et(Ind)_2_ZrCl_2_/MMAO catalytic system (Figure 5e). Copolymers with POSS-*i*-Bu-3 were characterized by a higher amount of silsesquioxane units in comparison with propylene/POSS-Cy-2 copolymers. Thermal stability of propylene copolymers with POSS was improved (by 5 to 40 °C in the case of *T_d_*) in comparison with neat polypropylene.

In this regard, there were no comprehensive studies on the influence of the structure of alkenylsilsesquioxane comonomers on the performance of copolymerization with olefins and physicochemical properties of copolymers, which limits the possibilities of planning and controlling the synthesis of copolymers with specific properties. Furthermore, there were no studies concerning the use of copolymerization of ethylene with multi-alkenylsilsesquioxanes. On the other hand, POSS units can improve thermal stability, what is a desirable effect in the case of polyolefins. Therefore, the purpose of our research was the comprehensive synthesis and the evaluation of composition and physicochemical properties of new hybrid ethylene copolymers with alkenylsilsesquioxanes, including different structure of the POSS cage. It was also important to select an active catalytic system in this process and to determine the optimal conditions for carrying out polymerization (including POSS concentration, amount of cocatalyst and catalyst, and reaction time), enabling the most effective synthesis of polyolefins containing alkenylsilsesquioxane units.

**Figure 6:**
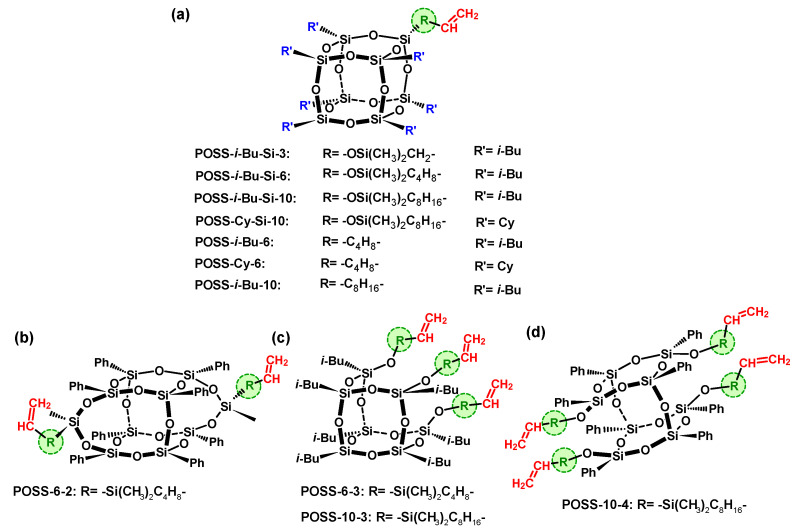
Structures of silsesquioxanes containing: monoalkenyl (**a**), dialkenyl (**b**), trialkenyl (**c**), tetraalkenyl (**d**) reactive substituents [71,72,73,74,75,76,77,78,79].

Low pressure *coordination copolymerization* of ethylene with alkenylsilsesquioxanes was described in [71,72,73,74,75]. The POSS comonomers used differed in structure, including the kind of silicon–oxygen POSS cage, type, and number of reactive, as well as non-reactive, substituents (Figure 6). In the case of monoalkenylsilsesquioxane, the structure of the reactive substituents in the silicon–oxygen cage differentiated the length of the hydrocarbon chain connecting the vinyl group with the POSS cage, as well as the presence or absence of a dimethylsiloxane bridge (-OSi(CH_3_)_2_-). Moreover, monoalkenylsilsesquioxanes contained *iso*-butyl (*i*Bu) or cyclohexyl (Cy) as the non-reactive functional group (Figure 6a). Furthermore, POSS with a different structure, including completely condensed (Figure 6b) and incompletely condensed POSS cage (Figure 6c,d), containing two, three, and four reactive alkenyl groups and *i*Bu or phenyl (Ph) as non-reactive groups, were used for the first time in copolymerization with ethylene.

**Figure 7:**
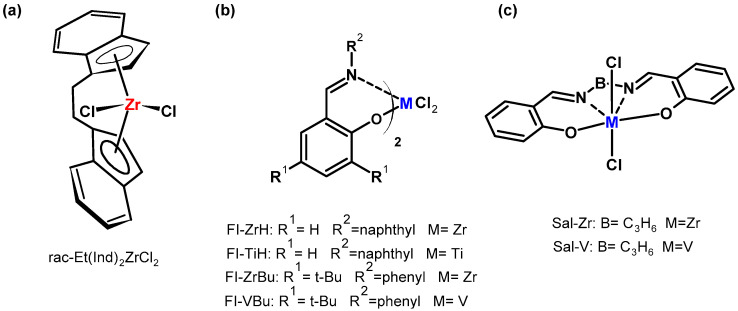
Structures of complexes: *ansa*-metallocene (**a**), bis (phenoxyimine) (**b**), salen type (**c**) used in copolymerization of ethylene with POSS [71,72,73,74,75].

A metallocene complex (Figure 7a) and, for the first time, post-metallocene catalytic systems were applied in copolymerization of ethylene with POSS (Figure 7b,c).

### 3.1. Ethylene Copolymerization with Mono-Alkenylsilsesquioxanes

It was demonstrated that the structure of POSS comonomers is one of the key factors determining the performance of ethylene copolymerization with POSS over *ansa*-metallocene catalysts (Figure 7a). Among the tested POSS derivatives, with *i*Bu groups as non-reactive substituents and different lengths of the reactive alkenyl substituent: POSS-*i*-Bu-2, POSS-*i*-Bu-3, POSS-*i*-Bu-6 and POSS-*i*-Bu-10 (Figure 6a), the comonomer content incorporated into the polymer chain was highest in case of copolymers of ethylene with POSS-*i*-Bu-6 [71]. This phenomenon was explained by the lower influence of both the steric and inductive effects of this substituent in the POSS comonomer on the ability to incorporate POSS into the polymer chain. It also turned out that appropriate selection of POSS comonomer results in the possibility of obtaining copolymers with diverse structures. Monofunctional POSS can be incorporated into the polymer chain in different ways, at the ends and internal macromolecules, depending on the presence or absence of a dimethylsiloxane bridge functioning as a connector of alkenyl substituent with POSS cage [71]. POSS incorporation degree was higher in the case of ethylene/monoalkenyl(siloxy)-POSS copolymers, in contrast to copolymers of ethylene with monoalkenyl-POSS, and fell within 0.10–3.15 wt.% and 0.07–2.32 wt.%, respectively.

The incorporation degree of POSS units into the polymer chain affected the thermal stability of the obtained copolymers [76]. The E/POSS copolymers had higher thermal stability in non-isothermal conditions, in comparison with neat polyethylene, regardless of the incorporation degree. In addition, POSS comonomers were characterized by much lower thermal stability in comparison with both E/POSS copolymers and neat PE, which was related with unique synergic influence of the polyethylene sequence and the POSS units. It was found that carefully selected crystallization condition of E/POSS copolymers resulted in improved thermal stability by several dozen °C for *T_5%_* (the temperatures at which 5% of the sample mass is lost) in comparison with crude copolymers [76]. Moreover, long-term heating of ethylene copolymers with monoalkenylsilsesquioxanes at static conditions (in constant temperature) allowed to both determine the structural changes in POSS-containing polyolefin materials during their thermal degradation and to propose a mechanism of these changes [76]. It was found that decomposition of E/POSS copolymers was dependent on the kind and content of POSS comonomer incorporated into the polymer chain. A low incorporation degree of silsesquioxane units incorporated into the polymer chain caused accelerated thermal degradation process of the products. In turn, copolymers with high POSS content were characterized by high thermo-oxidative stability in comparison with neat polyethylene. Ethylene/POSS copolymers with dimethylsiloxy bridges in reactive substituent were characterized by slower rates of thermal-oxidative degradation than products without bridges.

Our investigation also confirmed the efficiency of post-metallocene catalysts (with phenoxy-imine and salen ligands) in copolymerization of ethylene with selected POSS comonomers [75]. Three bis(phenoxyimine) complexes (FI catalysts) were used with different types of metal active centers (titanium and zirconium); the naphthyl group is *R*^2^ and with a hydrogen atom, or the *tert*-butyl group as *R*^1^ substituents in phenoxy-imine ligands (Figure 7b). Moreover, salen-type complex of zirconium (Figure 7c) was also tested. Ethylene was copolymerized with selected *iso*-butyl-substituted monoalkenyl(siloxy)silsesquioxane (POSS-*i*-Bu-Si-6 and POSS-*i*-Bu-Si-10, Figure 6a) and monoalkenylsilsesquioxane comonomers (POSS- *i*-Bu-6 and POSS- *i*-Bu-10, Figure 6a). Ethylene copolymers with monofunctional POSS synthesized by postmetallocene catalysts were characterized by a significantly higher incorporation degree into the polymer chain (0.14–6.40 wt.%) than those produced by *ansa*-metallocene (0.07–3.15 wt.%), a result which has been associated with the different structural parameters of postmetallocene catalysts [75] in comparison with *ansa*-metallocene catalyst [71]. Moreover, the structure of postmetallocene catalyst affected the incorporation degree of the POSS comonomer into the polymer chain and the properties of the obtained copolymers. The FI-ZrH complex turned out to be most effective during the incorporation of POSS comonomers into the polymer chain among the bis(phenoxyimine) complexes studied (Figure 7b). However, copolymers with the highest POSS contents in E/POSS copolymers were synthesized by the Zr–salen complex (Figure 7c). These results were explained by the fact that the active site of salen-type complexes is sterically more “open” for the insertion of the comonomer, in relation to the bis(phenoxyimine) catalysts.

Moreover, it was also found that the thermal stability of E/POSS copolymers obtained by bis(phenoxyimine)zirconium complex was higher at the similar POSS content in the polymer chain than that of the products obtained using the *rac*-Et(Ind)_2_ZrCl_2_ (Figure 7a). This phenomenon has been explained by different internal structures of E/POSS evaluated over WAXS (wide-angle X-ray diffraction) and SAXS (small-angle X-ray scattering) studies [75]. The WAXS and SAXS investigation of E/POSS copolymers also showed that the self-aggregation of POSS incorporated into the polymer chain was limited, and, therefore, the number and the size of the POSS aggregates were very small. Additionally, it was found that the POSS units as pendant groups of the polymer chain were most probably located in the amorphous regions outside the lamellar stacks.

### 3.2. Ethylene Copolymerization with Di-, Tri- and Tetra-Alkenylsilsesquioxanes

In case of copolymers of ethylene with di-, tri- or tetra-alkenylsilsesquioxanes, incorporated POSS units can constitute a side branch of the main polymer chain or cross-linked structure, where alkenylsilsesquioxane acts as a cross-linking agent of macromolecules, which is unusual for ethylene/POSS copolymers [72,73]. The di- and tetra-alkenylsilsesquioxanes (Figure 6b,d) can be incorporated into the polymer chain as a pendant group and can also act as a crosslinker of macromolecules, dependent on the reaction condition. The ethylene/di-alkenyl POSS and ethylene/tetra-alkenyl POSS with cross-linked structures were obtained in the case of low ethylene pressure in the polymerization feed (*p_e_* = 0.2 MPa), whereas higher ethylene pressure (*p_e_* = 0.5 MPa) resulted in synthesizing copolymers with linear structures. In turn, the tri-alkenylsilsesquioxanes (Figure 6c) were incorporated into the polymer only as a side branch of the main polymer chain, the same as monoalkenylsilsesquioxanes (Figure 6a), regardless of the polymerization conditions. It also turned out that the E/POSS-6-2 copolymer was characterized by the highest comonomer content among the investigated E/POSS copolymers. Moreover, the difunctional POSS derivative was characterized by a much higher reactivity (lower relative reactivity of ethylene 24.0), compared to monoalkenyl- and monoalkenyl(siloxy)silsesquioxanes, where the relative reactivity of ethylene was higher and ranged from 29.8 to 222.1.

An important part of the research was the study of thermal resistance (in dynamic conditions in air atmosphere using TG analysis) of ethylene copolymers with multi-alkenylsilsesquioxanes (di-, tri- and tetra-alkenyl POSS) produced by organometallic catalysts and the relation between the copolymer structure and its physicochemical properties [77,78]. It was found that thermal stability of E/POSS copolymers was dependent on the way of POSS incorporation into the polymer chain, which determines the type and content of unsaturated groups in macromolecules. It was demonstrated that the increase in trisubstituted vinylene groups significantly decreases the thermal stability of E/POSS copolymers [78]. Homogeneity of copolymers was found to be an additional factor affecting thermal stability. As demonstrated, higher homogeneity of polymer products causes higher thermal resistance. Moreover, cross-linked ethylene copolymers with POSS contained incompletely condensed silicon–oxygen cores and were characterized by the lowest thermal stability of all the materials studied with completely condensed POSS cages.

In addition, the thermal degradation of ethylene copolymers with di-, tri- and tetra-alkenylsilsesquioxanes was evaluated in detail under isothermal conditions (100 °C) based on structural changes in FT-IR spectrum [77]. The kind and content of multi-alkenylsilsesquioxane units incorporated into the polymer chain, as well as the structure of macromolecules, significantly influenced thermal stability of obtained copolymers. Incorporation of di-alkenylsilsesquioxane with a completely condensed POSS cage into the polymer chain caused significant improvement of thermal stability. Incorporation of POSS-6-2 as a side branch of the main chain and as a crosslinker of macromolecules resulted in a decreased reaction rate of the thermo–oxidative degradation process of copolymers in comparison with neat PE. High thermal stability in iso-thermal conditions of crosslinked E/POSS-6-2 copolymers is associated with high strength of the rigid polymer network at a low temperature range (100 °C) in contrast to the non-isothermal condition in TGA [77]. In turn, ethylene copolymers with POSS-10-3 or POSS-10-4 comonomers with incompletely condensed silicon–oxygen POSS cages were characterized by high susceptibility to thermal–oxidation degradation and turned out to be copolymers with much lower thermal stability in comparison with ethylene/monoalkenyl-POSS copolymers [76,78]. It should be highlighted that the key role in thermal degradation of E/POSS copolymers during their heating at a constant temperature was mainly played by the type of silicon–oxygen cage contained in the POSS unit, and also the structure of macromolecules of E/POSS copolymers.

One of the most interesting results of the research on ethylene/POSS copolymerization was demonstrating that tri-alkenylsilsesquioxane containing an incompletely condensed POSS cage not only acted as a comonomer but also transformed the inactive forms of the *ansa*-metallocene centers into precursors of the centers of active forms [72,74]. Modification of inactive forms of centers to active by the tri-alkenylsilsesquioxane comonomer was observed even at very low molar ratios of methylaluminoxane/*ansa*-metallocene; additionally, it competed with the cocatalyst for access to the catalyst, which is an unprecedented phenomenon in case of coordination polymerization [72].

Moreover, the morphological, viscoelastic, and dynamic mechanical properties of ethylene/multi-alkenylsilsesquioxanes copolymers with different architecture (linear and cross-linked) were also investigated [79]. The WAXS and SAXS analysis of ethylene/POSS copolymers found that POSS unit incorporated into the polymer chain did not have an influence on the crystalline structure of lamellar, which confirmed that POSS was located in amorphous phase, as was the case with copolymers of ethylene with monoalkenyl- and monoalkenyl(siloxy)silsesquioxanes [75]. In turn, rheological investigation showed that neat PE and linear ethylene/multi-alkenylsilsesquioxanes copolymers behaved like a viscous liquid structure, in contrast to cross-linked copolymers, which were a viscoelastic gel [79]. Linear E/POSS copolymers behaved as a lubricant due to the presence of high steric hindrance in the copolymer chain, in contrast to cross-linked products. In addition, the incorporation of POSS into the polymer chain as a pendant group resulted in a significant reduction of the relaxation time of the reputation process. Moreover, the structure of macromolecules markedly influenced the mechanical properties of the obtained copolymeric products [79]. Linear E/POSS copolymers were characterized by a lower value of the storage modulus in comparison with neat PE, in contrast to cross-linked copolymers. Moreover, a significant influence of the cross-linking process of di- and tetra-alkenylsilsesquioxanes on the improvement of the mechanical properties of the obtained E/POSS copolymers was found.

## 4. Silsesquioxane Derivates as Fillers in Composites Based on Polyolefins

As already mentioned, significant growth in technology over the last few decades has created the need to design and fabricate new materials with improved, and sometimes new, properties. Recent trends in this area are related to the development of hybrid composite materials. Introduction of inorganic substances as fillers into polymer matrices improved selected properties of polymer materials, such as stiffness, breaking strength, hardness, thermal stability, flammability, etc., and sometimes reduced their costs [80]. The most commonly used inorganic fillers include primarily chalk (CaCO_3_), talc, metal oxides (TiO_2_, ZnO, Al_2_O_3_, Fe_2_O_3_, SiO_2_), metal hydroxides (Mg(OH)_2_, Al(OH)_3_), layered silicates (montmorillonite, smectite, mica), and others [80,81,82,83,84,85]. In the group of inorganic fillers, silicon compounds, and particularly silica, gained a special importance [86]. This is mainly due to their low price and general availability, as well as their high thermal stability and favorable physicochemical properties [86]. Unfortunately, silica from natural resources (quartz, tridymite, cristobalite) is contaminated with metals, which limits its use [87]. For this reason, synthetic silica obtained by the sol-gel method (the so-called Stöber method) has become attractive [82,84,88,89]. Moreover, using the sol-gel method enables its functionalization. Introduction of appropriate functional groups on the silica surface reduces the tendency of this hydrophilic filler to aggregate in the hydrophobic polymer matrix [86,90,91,92,93]. Polyhedral oligomeric silsesquioxanes are another interesting alternative for silica due to the possibility of modifying their structure in a wide range [3,5,8,94,95,96,97]. Moreover, POSS compounds, as three-dimensional networks with a silica-like core covered by an organic coating, and particle size in the range of 1–3 nm, are also considered to be a promising alternative to other nanofillers such as organoclays, nanosilicas, or carbon nanofillers and nanotubes. Thus, recently, much attention has been paid to polymer nanocomposites containing POSS as fillers and different polymer matrices, among which the most important are commercially used petroleum-based polymers, such as polyolefins, poly(vinyl chloride), poly(methyl methacrylate), polystyrene, polyesters, epoxy and phenolic resins, polyurethanes, or polyamides, as well as biodegradable polymers, mainly polylactide. Significant results from this area have already been discussed in several literature reviews [6,57,98,99,100,101,102,103,104] and some of them also describe polyolefin/POSS nanocomposites [6,57,99,101].

### 4.1. Polyolefin/POSS Nanocomposites

Over the past two decades, there has been a rapid increase in research interest in exploring POSS-containing polyolefin nanocomposites. As the polyolefin matrix, isotactic polypropylene (*i*PP), different grades of polyethylene (including high-, low- and linear low-density polyethylene (HDPE, LDPE, and LLDPE)), and ultra-high molecular weight polyethylene have been used. Silsesquioxanes applied as fillers were structurally very diverse, with completely and incompletely condensed silicon cores, both monofunctional and multifunctional, including homo- and heterosubstituted compounds. POSS fillers were usually incorporated into the polyolefin matrix through convenient and simple melt blending. Nonreactive POSS molecules were mixed with polymer using an array of extruders and internal mixers. The amount of POSS fillers introduced into the polyolefin matrix usually does not exceed 10 wt%, and most often it is below 1 wt% and even parts per million [105,106]. The higher share of POSS caused problems with miscibility between POSS additives and the polyolefin matrix. This is due to the aggregation of filler particles and the heterogeneity of the polymeric material, and results in the deterioration of its properties [107]. Since POSS compounds can be easily functionalized by chemically altering the substituent groups at the silicon cage, this made them more attractive fillers for composites based on hydrophobic polyolefins. Therefore, the most frequently selected POSS moieties were those with a fully condensed T8 cage and eight nonreactive substituents attached to each silicon corner, mainly alkyl groups, but also vinyl and phenyl substituents.

The impact of POSS additive on the crystallization behavior of the polyolefin matrix inter alia was studied in the first paper describing the use of nanostructured POSS moiety as a filler of *i*PP nanocomposites [108]. The chosen filler, octamethyl-POSS, was added by melt blending to *i*PP at quite large concentrations (10 to 30 wt%). POSS particles were found to influence the crystallization process of *i*PP under both isothermal and nonisothermal conditions by acting as nucleating agents. Moreover, despite the significant share of the filler used, formation of its aggregates in the matrix was not observed. In turn, other researchers [109] studying morphology of polypropylene nanocomposites containing the same filler in an amount even below 10 wt% found poor miscibility between the polymer and the POSS compound. They also showed that increasing alkyl substituent length from methyl to isobutyl improved the compatibility between POSS and PP; however, further increase in the substituent length to *i*-octyl did not bring any apparent improvement of POSS dispersion. On the other hand, Chen et al. found [110] that POSS molecules aggregating to form nanocrystals due to shorter chain length of functionalized methyl substituents in the POSS cage act as an effective nucleating agent for *i*PP and affect the thermal properties of *i*PP/POSS nanocomposites.

The influence of POSS substituent groups on morphological and thermal characteristics of melt-blended POSS/*i*PP composites covered mainly the properties of nanocomposites, which contained POSS with relatively short alkyl chain substituents (from methyl to *i*-octyl) [108,109,110,111,112,113,114,115,116,117]. However, much longer alkyl chains reducing the hydrophilic nature of filler particles would enhance their compatibility with polyolefin chains, and hence the level of dispersion of POSS nanofiller in the polymer matrix and, finally, the expected physicochemical properties of the obtained materials. Heeley et al. [118,119] synthesized a unique set of T8 POSS compounds with long linear alkyl-chain substituents where the chain length was C_n_H_2n+1_, and *n* = 8, 12 and 18. The selected POSS compounds were blended with low density polyethylene. They reported that the miscibility and dispersal of the POSS molecules was seen to increase with the increasing alkyl-chain length substituents, suggesting increased compatibility and interaction with host polymer chains.

Based on this assumption in our studies [120,121,122,123], T_8_ POSS with eight *n*-octyl and *n*-octadecyl substituents were melt blended in various contents (1–10 wt%) with commercially available polyolefins (*i*PP, LDPE and HDPE), and the influence of the structure and the content of POSS nanofiller into the matrix on the morphology and properties of the obtained nanocomposites were assessed. It was established that the properties of polyolefin/POSS nanocomposites depend mainly on the structure of the *n*-alkyl groups attached to the silicon–oxygen POSS core. Longer alkyl chains in the filler molecule improve its compatibility and dispersion in the material, which was confirmed based on the results of structural properties tests using scanning electron microscopy and X-ray energy dispersive spectroscopy (SEM/EDS), WAXS, SAXS, position annihilation lifetime spectroscopy (PALS), and decomposable entropic descriptor methods [122]. Moreover, the effect of POSS particles as effective nucleating agents improving the crystallization process for polyolefin matrices was clearly demonstrated. POSS compounds containing shorter (*n*-octyl) hydrocarbon substituents in their structure proved to be more effective as agents which accelerate the polymer crystallization process [121,122]. The performed studies also revealed that obtained polyolefin/POSS nanocomposites are generally characterized by improved thermal, mechanical, and processing properties, in comparison with neat polymers [122]. Moreover, they showed that increasing the length of hydrocarbon chains from *n*-octyl to *n*-octadecyl in silicon filler molecules resulted in improved mechanical and processing properties, as well as in higher thermal stability and reduced flammability of polyolefin materials. Importantly, the presence of long hydrocarbon chains in the filler molecules had a key role in providing their high homogeneity in the polymer matrix, and thereby ensuring the appropriate reinforcement of such materials [122,123].

Not so long ago, Zhang et al. reported [124] that a novel long-chain alkyl (C30-45) modified silsesquioxane, namely (C30-45 alkyl) dimethylsilyl polypropylsilsesquioxane (C30PSS), was applied as a functional filler for linear low-density polyethylene. For comparison, LLDPE/octa-*i*-butyl polyhedral oligomeric silsesquioxane (BuPOSS) nanocomposites were also prepared via melt blending. Dispersion of two fillers, C30PSS and BuPOSS, in the polymer matrix was analyzed by the SEM/EDS method. The properties of neat LLDPE, LLDPE/C30PSS, and LLDPE/BuPOSS composites were studied, and the obtained results showed that C30PSS could be used as an inexpensive filler providing good dispersion in the LLDPE matrix and improving the rheological and mechanical properties of the polymeric material.

A special group of silsesquioxane fillers of polyolefin-based nanocomposites are metal-containing POSS, initially interesting for their potential catalytic activity and later also investigated as additives for modifying structural, morphological, and thermal properties of the polyolefin matrix. Fina et al. investigated dimeric and oligomeric Al and Zn-containing *i*-butyl silsesquioxanes [125,126], and Carniato et al. used monomeric *i*-butylsilsesquioxanes with Ti^IV^, V^V^ and Al^III^-metal centers as fillers in polypropylene nanocomposites [127,128]. The influence of the nature of these metal–POSS compounds and the presence of different metal centers in the siliceous framework on the final properties of the nanocomposite materials were studied. It was found that the dispersion of metal–POSS filler in polypropylene-based composites obtained by a melt-blending process was very successful, and that POSS compounds, due to their chemical behavior, allow it to obtain materials with higher thermal stability and higher fire resistance, thus enlarging the application field of organic polymers.

Previous studies of composites based on polyolefins with POSS fillers, differing in the structure of the silicon–oxygen cage, and, above all, in the type and number of substituents, have shown that the incorporation of heat-resistant POSS compounds into the polyolefin matrix affects the crystallization and melting characteristics of the polymer; modifies its thermal properties, providing greater thermal stability; improves mechanical properties, such as tensile or flexural modulus, tensile strength, heat deflection temperature, and rheological properties of materials (e.g., as plasticizers or thixotropic agents); and even reduces flammability of the obtained polymer material. It is believed that the observed effects result mainly from the POSS–polymer and POSS–POSS interactions, which depends on the structure of the substituents attached to the POSS cage.

POSS compounds were also introduced as an additional component to polymers already containing another functional additive to enhance its function. The synergistic effect of both additives allowed to enhance the improvement of the desired properties of the composites using a very small amount of POSS, which is important for practical use considering the high price of silsesquioxanes. Roy et al. [129,130,131] and other researchers [132,133] reported the effect of chemical combination of POSS compounds with sorbitol on the crystallization, rheological, and mechanical properties of *i*PP nanocomposites. It was stated that incorporation of POSS–sorbitol into *i*PP could significantly increase the crystallinity and the mechanical properties of the polymer material. In turn, Mao et al. reported [134] that an absorbed *β*-nucleating agent on the surface of POSS not only improved the interfacial interaction between POSS and the polymer matrix using interfacial crystallization, but also promoted formation of β-crystals with a toughening effect, which is very important to prepare excellent-performance PP/POSS nanocomposites and to reduce the amount of expensive POSS compound used. Dintcheva et al. [135] synthesized multifunctional filler based on carbon nanotubes (CNTs) and POSS with phenyl substituents (phPOSS), and 1 wt% was dispersed in UHMWPE in order to improve the photo-stability of resulting nanocomposite. The obtained multifunctional filler showed a remarkable protective ability, which was much higher than that exerted by CNTs and phPOSS molecules when added one by one. In turn, rheological and thermo–mechanical studies of nanocomposites demonstrated the reinforcement effect of CNTs and the plasticizing function of phPOSS molecules.

In addition to improving the mechanical performance and thermal properties of polyolefins, POSS molecules are introduced into these highly flammable polymers to also improve their flame retardancy. Zhang et al. recently reported [136] a flame-retardant PP composite, which consists of synthesized single component intumescent flame retardant (IFR) and the novel phosphorus-containing polyhedral oligomeric silsesquioxane (P-POSS). They found that only 1 wt% P-POSS can remarkably improve the fire retardancy of such a composite material, playing synergistic role with IFR. The addition of P-POSS also helped to enhance the mechanical performance of the obtained composite by improving interfacial compatibility and additive dispersion. In turn, the effect of four different POSS nanoparticles (aminopropyl isobutyl-POSS, octaphenyl-POSS, octaammonium-POSS, and trissulfonic acid propyl-POSS) on the flame retardant properties of ammonium polyphosphate/pentaerythritol (IFR) in PP composites was investigated by Turgut et al. [137]. All POSS-containing composites showed better fire performance than original IFR-containing composite.

### 4.2. Siloxane-Silsesquioxane Resins as Fillers of Polyolefin Nanocomposites

**Figure 8:**
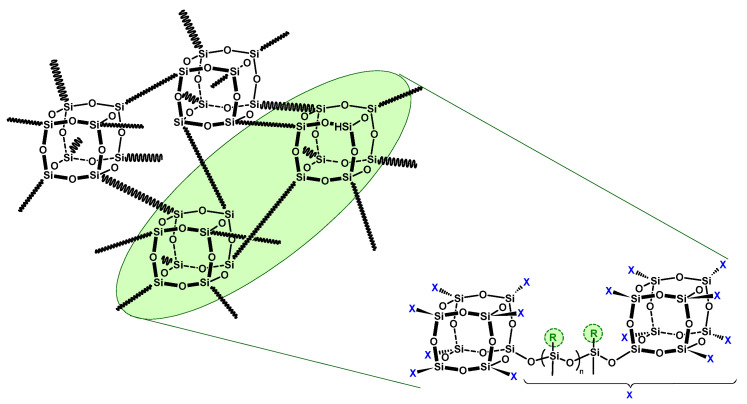
Structures of S4SQ-R siloxane–silsesquioxane resins.

As mentioned above, despite a number of beneficial properties of polyolefin/POSS composites, their commercial use is still limited due to the high cost of POSS fillers. This contributed to the development of siloxane–silsesquioxane resins as a less expensive alternative, which may retain the advantageous features of typical POSS compounds. The siloxane–silsesquioxane resins are characterized by a unique extended network structure, in which the silicon–oxygen T_8_- or Q_8_-type cages constitute network nodes, and these are connected with each other by siloxane bridges. An important advantage of this group of compounds is the possibility to vary their structure by changing the length of the siloxane bridge and its functionalization by the introduction of appropriate functional groups [138]. Examples of the structures of such resins are shown in Figure 8.

Siloxane-silsesquioxane resins are relatively new materials, and, in the literature, there is a limited number of studies on synthesis, characterization, and possible applications of these compounds. In addition, only a few of them relate to composite materials with siloxane-silsesquioxane resins as fillers. Dobrzynska-Mizera et al. [139,140] reported polypropylene-based composites containing a phenyl-functionalized siloxane-silsesquioxane resin (SiOPh) and sorbitol derivatives. They established that the addition of SiOPh resin into PP enables control of the nucleation efficiency of sorbitol derivatives and allows to adjust the crystallization rate of PP due to the influence of SiOPh on the formation of the sorbitol fibrillar network. Recently, other researchers [141] have applied a series of siloxane–silsesquioxane resins, which were functionalized with hydroxypropyl groups and differed in the length of siloxane chains linking silsesquioxane Q_8_ nodes, for the preparation of polyurethane-based materials. In that case, siloxane–silsesquioxane resins were capable of bonding covalently with the polyurethane matrix. The resulting composite materials showed improved thermal stability or increased crystallization temperatures depending on the length of siloxane chains in their structure.

In our studies, non-functionalized (containing hydrogen atoms as substituents in siloxane bridges) and *n*-alkyl-functionalized S4SQ-8 and S4SQ-18 (with *n*-octyl and *n*-octadecyl groups, respectively) siloxane–silsesquioxane resins were synthesized and applied as fillers for polypropylene and polyethylene nanocomposites [142,143,144,145]. Polyolefin/S4SQ-R composites containing from 1 to 10 wt% of the appropriate resin were obtained by melt blending. The produced materials were subjected to comprehensive characteristics in order to determine the effect of the siloxane–silsesquioxane resins on the structural properties and, as a consequence, on the functional properties of the composites. Due to the crosslinked structure of the siloxane–silsesquioxane resins, their functionalization with *n*-alkyl groups determined the achievement of homogeneous composite materials, which was confirmed with the use of SEM/EDS and WAXS methods [142,143]. It was found, similarly to our previous studies of polyolefin/POSS nanocomposites, that functionalization of siloxane–silsesquioxane resins with long alkyl groups (S4SQ-8 and S4SQ-18) improved filler–polymer miscibility and, consequently, contributed to uniform dispersion of resin particles in the polymer matrix. In contrast, significant aggregation of filler particles was observed in the case of composites with non-functionalized S4SQ-H resin. The introduction of S4SQ-R particles into the polypropylene matrix resulted in a decrease in total crystallinity and an increase in the sizes of crystallites. Moreover, the 𝛽-nucleation process of polypropylene was hindered by the presence of siloxane–silsesquioxane resins in materials. Simultaneously, these filler particles increased the crystallinity degree of the PP *α*-phase. The average parameters of lamellar stacks (evidenced by SAXS data) and the changes in PALS parameters confirmed that alkyl-functionalized siloxane–silsesquioxane resins are located mainly in the amorphous phase of the polymer due to their highly extensive network structure. In turn, particles of non-functionalized S4SQ-H resin could be located both in the crystalline and amorphous regions. DSC analysis revealed that siloxane–silsesquioxane resins enhanced the crystallization process of PE and PP neat polymer, which proved that they could be considered as nucleating agents. Thermal, mechanical, and processing properties, as well as thermal stability and flammability, of the polyolefin/siloxane–silsesquioxane resin composites varied depending on the structure of the filler used. Moreover the presence of long hydrocarbon chains in the filler molecules had a key role in providing their high homogeneity in the polymer matrix, and thereby ensured the appropriate reinforcement of such materials. Materials containing non-functionalized siloxane–silsesquioxane resins were, in principle, characterized by the least favorable properties, despite the fact that they were often better in comparison with the properties of neat polymers.

Moreover, composites filled with siloxane–silsesquioxane resins showed a large similarity to materials containing alkyl-functionalized POSS. Silicon compounds containing shorter hydrocarbon substituents in their structures proved to be more effective as agents, which accelerated the polymer crystallization process. In turn, increasing the length of hydrocarbon chains in silicon filler molecules resulted in improved mechanical and processing properties, as well as in higher thermal stability and reduced flammability of polyolefin materials [144,145].

It could be stated, on the basis of our studies, that *n*-octyl and *n*-octadecyl functionalized polyhedral oligomeric silsesquioxanes and siloxane–silsesquioxane resins may be considered as promising nanofillers and also as UV stabilizers and/or antioxidants for polyolefin nanocomposites. However, it should be emphasized that resin particles turned out to be the more effective nucleants than POSS with the same substituents.

Finally, the results confirm that, through appropriate selection of the structure of silicon compounds, as well as the type and length of *n*-alkyl substituents in these molecules, it is possible to design the properties of polyolefin composite materials for a given direction of their use.

## 5. Conclusions

Application of organic–inorganic compounds such as polyhedral oligomeric silsesquioxanes and siloxane–silsesquioxane resins in the area of polyolefins opened up the door for preparation of materials with improved properties. Incorporation of these compounds to polymeric materials both as comonomers and as fillers has a beneficial effect on many properties of the obtained copolymers and composites, among which the most interesting are the crystallization behavior, higher thermal stability, and reduced flammability. Moreover, easy modification of silicon compounds by altering the structure of non-reactive and/or reactive substituents attached to the silicon–oxygen core enables not only to design polyolefin materials with a wide range of physico–chemical properties, but also makes silsesquioxanes applicable in the creation of new catalysts for olefin polymerization, including homogeneous analogs of supported catalysts. This allowed for a better understanding of the mechanism of ethylene polymerization with industrial Philips-type catalysts. On the other hand, this review showed the limitations of using silsesquioxanes in the synthesis of polyolefins. The limitations concern the activity of silsesquioxane complexes (much below the activity of many metallocene and postmetallocene catalysts) and their low comonomer incorporation capacity. Furthermore, until today, there have been no known catalytic systems that would be able to incorporate POSS-comonomer into polyethylene macromolecules with high efficiency. Finally, although much attention has been paid to the influence of the silsesquioxane structure on compatibility between composite components and the properties of the materials, the correlation between the microscopic and macroscopic properties of polyolefin/POSS nanocomposites, which is of particular importance for the design of their properties, has not been fully resolved so far. Thus, further investigations regarding the application of silsesquioxanes in polyolefin synthesis and modification are expected. As has been shown, applications of silsesquioxanes in the field of polyolefin are very diverse, and their use in each area significantly expands our knowledge about polyolefins. However, from a practical point of view, the use of silsesquioxanes as fillers, especially additives used in small amounts (due to the high price of POSS), for the desired modification of polymer properties with other modifiers seems to be the most promising. The use of much cheaper and effective siloxane–silsesquioxane resins seems particularly interesting for this purpose.

## Data Availability

No new data were created or analyzed in this study. Data sharing is not applicable to this article.
